# How Does the Sparse Memory “Engram” Neurons Encode the Memory of a Spatial–Temporal Event?

**DOI:** 10.3389/fncir.2016.00061

**Published:** 2016-08-23

**Authors:** Ji-Song Guan, Jun Jiang, Hong Xie, Kai-Yuan Liu

**Affiliations:** ^1^Ministry of Education Key Laboratory of Protein Sciences, School of Life Sciences, Tsinghua UniversityBeijing, China; ^2^IDG/McGovern Institute for Brain Research at Tsinghua University, School of Life Sciences, Tsinghua UniversityBeijing, China; ^3^Center for Brain inspired Computing, Tsinghua UniversityBeijing, China

**Keywords:** memory, trace neurons, memory allocation, immediate early gene, memory storage, recall, circuit

## Abstract

Episodic memory in human brain is not a fixed 2-D picture but a highly dynamic movie serial, integrating information at both the temporal and the spatial domains. Recent studies in neuroscience reveal that memory storage and recall are closely related to the activities in discrete memory engram (trace) neurons within the dentate gyrus region of hippocampus and the layer 2/3 of neocortex. More strikingly, optogenetic reactivation of those memory trace neurons is able to trigger the recall of naturally encoded memory. It is still unknown how the discrete memory traces encode and reactivate the memory. Considering a particular memory normally represents a natural event, which consists of information at both the temporal and spatial domains, it is unknown how the discrete trace neurons could reconstitute such enriched information in the brain. Furthermore, as the optogenetic-stimuli induced recall of memory did not depend on firing pattern of the memory traces, it is most likely that the spatial activation pattern, but not the temporal activation pattern of the discrete memory trace neurons encodes the memory in the brain. How does the neural circuit convert the activities in the spatial domain into the temporal domain to reconstitute memory of a natural event? By reviewing the literature, here we present how the memory engram (trace) neurons are selected and consolidated in the brain. Then, we will discuss the main challenges in the memory trace theory. In the end, we will provide a plausible model of memory trace cell network, underlying the conversion of neural activities between the spatial domain and the temporal domain. We will also discuss on how the activation of sparse memory trace neurons might trigger the replay of neural activities in specific temporal patterns.

## Introduction

Memory is essential for the everyday life of human beings, as well as animals. So far, it is still a challenging question to answer where and how the memories are stored in the brain. In the early days of neuroscience, memory was considered as a cognitive substance, which is closely related to the anatomical features of neurons. Cajal (1894) proposed that memories may be formed by strengthening the connections between specific neurons to enhance their communication efficiency. Hebb (1949) further extended this proposal, which was summarized as “Cells that fire together, wire together” (Lowel and Singer, 1992). The discovery of long-term potentiation (LTP) in synapses provides the potential physiological evidence for this hypothesis (Bliss and Lomo, 1973). By now, LTP is considered as one of the fundamental cellular mechanisms for memory storage in the brain (Bliss, 1990; Bliss and Collingridge, 1993; Maren and Baudry, 1995; Wang et al., 1997; Cooke and Bliss, 2006). Besides LTP, other forms of long-term synaptic plasticity, such as long-term depression (LTD), also involve in memory formation (Ito, 1986; Zhuo and Hawkins, 1995; Stanton, 1996).

With the application of advanced research tools in neuroscience, direct evidences gradually emerged and showed that LTP and synaptic plasticity were sufficient and required for the encoding and recall of memory, especially in fear memory (Morris et al., 1986; Davis et al., 1992; Whitlock et al., 2006; Monteggia et al., 2014; Hayashi-Takagi et al., 2015). In addition to the activity dependent change of synaptic transmission, neuromodulators also contribute to the plastic modulations of the neural network. Early works have shown neuromodulators, such as adenosine, dopamine, and acetylcholine, influence synaptic plasticity (Poorthuis et al., 2014; Puig et al., 2014; Sebastiao and Ribeiro, 2015). Neurotropic factors, such as brain-derived neurotropic factor (BDNF), have great impacts on synaptic plasticity (Coppens et al., 2011; Benekareddy et al., 2013). So far, the synaptic plasticity has been considered as the neuronal substrate of the memory. However, due to the complexity and dynamics of synapses within the brain, it is hard to locate all of the modified synapses during the encoding of a specific memory to locate and track the specific memory.

By now, it is still an open question how the neural circuit encodes a specific memory and recalls it later. In the past few years, accumulating evidence suggests that memory in the brain is closely related to the activities of a small population of neurons. The sparse and selective activities were found during memory encoding and recall in many areas within the brain, such as amygdala, the dentate gyrus (DG) region of hippocampus and the layer 2/3 of neocortex (Han et al., 2007; Liu et al., 2012; Ramirez et al., 2013b; Cowansage et al., 2014; Santoro and Frankland, 2014; Xie et al., 2014). Those selective neuron populations were named as memory traces or the “engram” in some circumstance. Activities of the memory trace cells are triggered by memory recall. Artificially activating those cells in an unrelated context or without the presence of cues could induce the expression of fear memory (Liu et al., 2012; Cowansage et al., 2014; Denny et al., 2014; Kim et al., 2014), indicating the memory trace cell circuit is critical for the memory encoding and recall. On the one hand, such approach greatly reduces the complexity of memory research by reducing the synaptic events into the cellular events for memory encoding and recall. On the other hand, as all of the studies as memory traces are based on the alternation of neuronal activities, according to the expression of immediate early genes, this approach lacks the precision of measurements in the temporal domain. Particularly, it is unclear how could the ensembles of sparse memory trace neurons encode the temporal sequence of neural activity to represent necessary components of the memory.

Here, we summarize the recent advances in the study of memory representation at the cellular level to give a rough picture about the memory encoding in the brain. We will also discuss the major challenges of the cellular encoding of memory. In the end, we will provide a model of the memory circuitry, trying to discuss the possibility of encoding and recall of temporal signals in the spatial domain of memory trace cells.

## What Is Memory Engram?

Semon (1921), a German zoologist and evolutionary biologist proposed the idea of “engram” of memory in the early 20th century. His main idea is how an external stimuli produces a “permanent record, … written or engraved on the irritable substance.” Different from the synaptic plasticity and memory theory, the memory engram theory put more efforts to elucidate the nature of selective memory contents in the brain circuit (Sutherland and McNaughton, 2000; Silva et al., 2009; Zhou et al., 2009; Sano et al., 2014; Josselyn et al., 2015). The general idea is that each memory should be associated with the activities in a distinct ensemble of neurons, so that activation of those cell ensembles triggers the recall of the specific memory. To demonstrate whether an ensemble of neurons are memory engram, they must satisfy the following four criteria: (i) their activities were increased during learning and underwent a serial molecular and functional cellular changes; (ii) artificially activating these neurons could lead to recall of originally formed memory, even without external stimuli; (iii) blocking their activity would prevent the recall of the established memory in spite of the stimulation of physiological relevant signals; (iv) these neurons are a small subset of the total population and are selectively activated by a distinct memory.

## Location of Memory Engram

To locate memory storage in the brain, Lashley (1950) conducted a series of brain lesion experiments by surgical destruction of parts of the brain in search of the location of memory engram in rats and monkeys. He trained rats with the maze task and found that if only a small part of cortex was destroyed, memory may remain almost intact. The larger amount of cortex were removed, the more severe memory defect would be observed. So he made the mass action conclusion that the amount of lost memory was proportion to the amount of cortex destroyed (Lashley, 1950). Furthermore, he proposed that memories were distributive in the neocortex, although he failed to identify the memory engram.

A strong link between medial temporal lobe (MTL) and memory was first discovered when memory defect of patient H.M. was reported (Scoville and Milner, 1957). After MTL lesion, H.M. unexpectedly developed profound amnesia. He was unable to form long-term episodic memories immediately after the surgery, while other kinds of memories such as motor skills and personality remained largely normal. The importance of MTL, mainly the hippocampal formation, in episodic memory was confirmed in both human and non-human primate and rodent in subsequent studies (Squire et al., 1975; Zola-Morgan et al., 1994; Rempel-Clower et al., 1996; Clark et al., 2002; Frankland and Bontempi, 2005). Conversely, it is unknown how the long-term memories, which were encoded long before the MTL damage, could still be retrieved. Thereby, there might be some alternative locations for the storage of long-term memory in the brain other than the hippocampal formation (Squire, 1992; McClelland et al., 1995; Squire and Alvarez, 1995; Nadel and Moscovitch, 1997; Qin et al., 1997; McGaugh, 2000).

In an effort to locate the memory-related activities in the brain, researchers switched their approaches from destroying subpopulation of the brain to tracking the neuronal activity changes in each brain region. With the development of molecular biology, researchers found that activation of neurons was accompanied by the expression of immediate-early genes (IEGs), such as *c-fos, Arc*, and *Egr-1.* Importantly, the expression of IEGs were triggered by learning new tasks (Cole et al., 1989; Guzowski et al., 1999; Guzowski and Worley, 2001; Wang et al., 2006; Flavell and Greenberg, 2008). By comparing the IEG expression signals during learning and memory retrieval, the brain regions activated by memory have been identified. The identified memory-related areas mainly include amygdala, DG of hippocampus and layer 2 of neocortex (Han et al., 2007; Liu et al., 2012; Ramirez et al., 2013a; Xie et al., 2014; Cao et al., 2015; Ramirez et al., 2015; Ryan et al., 2015).

Not until recently, genetic tools have been developed to label and manipulate those activated neurons in the history so that one could identify the reactivation of neurons in the same animal during memory recall (Reijmers et al., 2007; Silva et al., 2009; Garner et al., 2012; Kim et al., 2014; Tonegawa et al., 2015b). Furthermore, such genetic tools allow the *in vivo* imaging of neuronal activation in living animals. The repetitive imaging of the IEG expression in the same animal put this effort forward to discriminate activities in several different tasks (Xie et al., 2014; Cao et al., 2015). These studies extended the specificity of memory trace activities at further refined spatial and temporal domains. Furthermore, the *in vivo* imaging of IEG expression revealed the laminar differences of memory responses in neocortex and identified memory trace cells in layer 2 of neocortex (Xie et al., 2014).

**FIGURE 1:**
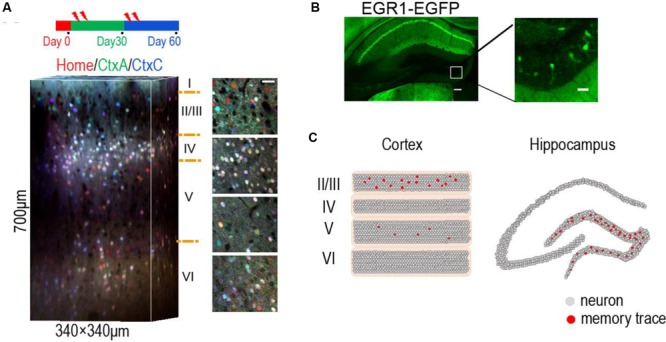
**Location of sparse memory trace cells in DG and layer 2/3 of neocortex. (A)** Representative 3-D reconstruction showing expression of EGR1-EGFP in three trials in different contexts (day 0, day 30, and day 60) from L1 to L6 in mouse visual cortex. EGFP signals in each trial were indicated by pseudocolor. Neurons in layer 2/3 show context specific response. Red home cage; green CtxA; blue CtxC. Ctx, Context. Volume size, 340 μm × 340 μm × 700 μm. Scale bar 30 μm. Reproduced from (Xie et al., 2014). **(B)** Representative images showing expression of EGR1–EGFP in hippocampus after enrich environment (EE) for 2 days. Right is an image of the rectangular area in the left panel, showing sparse activation of EE memory trace cells in DG. Scale bar: left, 100 μm; right 20 μm. **(C)** The schematic diagram shows memory engram cells in layer 2/3 of neocortex (left) and DG area of hippocampus (right).

Optogenetic and chemical genetic tools allow the manipulation of neural activities and further confirmed the role of memory engrams through artificially induced memory recall (Zhang et al., 2006; Gradinaru et al., 2008; Liu et al., 2012; Guenthner et al., 2013; Kim et al., 2014; Yiu et al., 2014). Such tools also provide a unique approach to dissect the circuit mechanisms for the memory-related activities. Thus, by applying IEG-based strategies, the researchers are now linking the memories with the sparse activities of specific cell ensembles in the brain (Tonegawa et al., 2015b) (**Figure 1**).

## The Allocation of Memory Engram for Different Tasks

Among huge number of neurons in the brain, only a small portion of neurons would be activated upon a given task (Wang et al., 2006; Han et al., 2007; Guenthner et al., 2013; Cao et al., 2015). For example, in amygdala, the central processor of fear memory (LeDoux, 2000; Fanselow and Gale, 2003; Rumpel et al., 2005), only a small fraction of neurons showed selective response to the cued fear (Rumpel et al., 2005; Han et al., 2007).

Consistent with the synaptic plasticity and memory theory, the learning-induced activity triggers synaptic plasticity and prolonged adaptation in those activated neuron ensembles. Cells with relative higher level of CREB (adenosine 3′, 5′-monophosphate response element-binding protein) in lateral amygdala (LA) were preferentially activated during the retrieval of cued fear memory (Han et al., 2007). Furthermore, selectively deletion of those CREB overexpressing neurons, but not randomly selected neurons, disrupted the recall of fear memory (Han et al., 2009). The similar results were obtained in insular cortex during memory retrieval for conditioned taste aversion task (Sano et al., 2014). Importantly, after cued fear conditioning, reactivation of those CREB overexpressing neurons through chemical genetic manipulation induced robust fear responses in the absence of external cue (Kim et al., 2014), indicating the CREB expressing neurons as the memory traces to encode and reactivate the memory. These results suggest a mechanism for memory allocation in memory trace neurons, which were prepared with high level of synaptic plasticity. Taken together, these results indicate the role of plasticity regulators, such as CREB protein (Davis et al., 1996; Deisseroth et al., 1996; Murphy and Segal, 1997; Bito, 1998; Schulz et al., 1999), are critical for the formation of memory traces in the brain circuit.

The expression of CREB increased the excitability and the efficacy of activity-induced synaptic changes, thereby allowing the encoding-related activities to be located in those cell ensembles (Zhou et al., 2009). Increasing cell excitability by expression of dominant-negative KCNQ2 mutant could also assign memory into those infected neurons in LA (Yiu et al., 2014). Apart from the over-expression of CREB, chemical genetic tools, such as DREADD (designer receptors exclusively activated by designer drug), an evolved G protein-coupled receptor (hM3Dq; Alexander et al., 2009), also attracted the formation of memory traces in those hM3Dq expressing neurons under synthetic ligand CNO induction (Garner et al., 2012).

Besides the fear memory, such pre-conditioning of memory trace location has also been demonstrated in spatial memory tasks. Hippocampal place cell was specifically activated at a certain location during the exploration of space (Hollup et al., 2001a,b; Brun et al., 2002; Moser et al., 2008). The experience of spatial exploration in a linear track triggered the firing of a serial of neurons under specific temporal sequence (Wilson and McNaughton, 1993; Lever et al., 2002; Hsiang et al., 2014). Interestingly, the firing pattern of place cell ensemble during exploration of a novel track had been occurred in the sleep period proceeding to the exploration (Dragoi and Tonegawa, 2011, 2013). Such observation suggests place cells might work in a pre-conditioned circuit to incorporate the spatial memory. Besides the place cells, time cells, which fire at particular moments in a temporally structured period, are observed recently in hippocampus (Manns et al., 2007; Naya and Suzuki, 2011; Eichenbaum, 2014), implicating that both the locations and the time components of the episodic memories are represented in hippocampus. Taken together, the memory traces for a specific memory are allocated to neurons of a heterogeneous population. The allocations of different memory traces are subjected to the regulation of the learning period through pre-conditioning mechanisms. Neurons with relatively higher excitability in the learning period are more inclined to encode the incoming information.

## Artificially Manipulation of Activities in Memory Traces Trigger Memory Recall

Upon the formation of memory traces, the reactivation of those trace cells is closely related to the memory recall. Firstly, the expressions of IEGs in those memory trace neurons were reactivated during the recall of memory (Han et al., 2007; Tayler et al., 2013). Secondly, genetic labeling of those memory traces, which expressing the IEG during learning period, revealed the selective activation of those trace neurons by specific memory, but not other stimuli (Denny et al., 2014). Lastly, optogenetic reactivation of those memory trace neurons engaged the memory recall in an unrelated context (Liu et al., 2014). As the expression of IEGs are positively correlated to the neural firing (Cole et al., 1989; Dragunow and Faull, 1989; Rakhade et al., 2007), those results showed that the activities within the memory trace neurons were critical for the memory recall.

Liu et al. (2012) labeled a group of DG neurons with channelrhodopsin-2 (ChR2) whose activities were increased during fear conditioning. They took the advantage of c-fos-tTA transgenic mice (Reijmers et al., 2007) to control the timing of genetic labeling. They labeled the *c-fos* expressing neurons during learning by removing the doxycycline. Without doxycycline, the activity-induced tTA protein, which is under the promoter of *c-fos*, could bind to tetracycline-responsive element (TRE) site, which in turn drives the expression of ChR2-YFP in these activated cells. The activities in these cells were controlled by blue light after genetic labeling. This strategy is able to trap the neurons in any brain areas activated during fear memory or happy memory, both of which were selectively reactivated by light in the unrelated context (Redondo et al., 2014). Optogenetically silencing the IEG-labeled neurons in DG (Denny et al., 2014) or CA1 (Tanaka et al., 2014) blocked fear memory recall, when re-exposing the mice into the shock associated context. Due to the specificity of those neurons, researchers named those cells as memory engram or memory trace cells.

Besides the engram cells in hippocampus, reactivation of memory trace cells in neocortex also triggers the recall of memory. Ensembles of neurons responding to training context were sparsely distributed in layer II and the activities of these neurons were linearly correlated with behavior (Xie et al., 2014). Optogenetically activating the learning-activated neurons in retrosplenial cortex (RSC) elicited fear memory (Cowansage et al., 2014). Furthermore, silencing of CA1 neurons, which were activated during learning, significantly reduced the reactivation of memory responses in neocortex and amygdala (Tanaka et al., 2014), indicating the memory traces in hippocampus and neocortex were closely correlated. Intriguingly, artificially activating memory engram cells could restore memory even under disease conditions, such as drug-induced retrograde amnesia and Alzheimer’s disease (Ramirez et al., 2015; Ryan et al., 2015; Roy et al., 2016). Thus, the activation of memory trace neurons induces memory recall.

## Challenges of The IEG-Labeled Memory Engram Theory Underlying Memory Encoding

Although the memory engram (trace) theory has been greatly improved by the recent progresses, several questions still remain to be answered. One of the questions is the precision of the labeled memory engram cells. So far, the memory engrams were identified according to their activities during memory task by the expression immediate early genes. However, due to the limitation of temporal precision of current genetic tools, this approach is not able to mark the memory-activated neurons very specifically. As the time window for genetic labeling is ranged from several hours to days (Clem et al., 2008; Matsuo et al., 2008; Alexander et al., 2009; Grinevich et al., 2009; Koya et al., 2009; Sakaguchi and Hayashi, 2012), most of the labeled memory trace neurons are, in fact, a mixed population, which contains both ‘memory trace cells’ for the specific memory and neurons activated by un-related events which occurred during the labeling window. To achieve higher precision on cell manipulation, one might need to continuously track cellular activities in living mice and selectively activate those identified trace neurons at cellular precision. Therefore, the issue, regarding the precise control of the population labeling during the learning period is solvable in the near future.

However, a much harder question is raised by the identification of those memory traces. The fact that reactivation of sparse memory engrams triggers the recall of memory, posts a theoretical question how the activities in a few neurons induce the representation of a memory, which contains well organized information under a specific temporal and spatial order. The transformation from sparse and simple activities in a few memory trace neurons to the wide spreading and temporally ordered activities in the brain network does not have a straightforward answer.

Strikingly, the reactivation of memory recall did not require temporally coded activities in the memory traces, as artificially imposing 20 Hz activities in those cells engaged the memory recall (Liu et al., 2012). This observation implicates that the neuronal network utilized the spatially organized structures and spatial activities in the memory trace neuron ensembles to encode and recall the natural event. Such encoding strategy used the spatial information to reconstruct information in both the temporal domain and the spatial domain. In the following sections, we put forward a model, trying to propose a possible solution of neural network to interpret the role of memory traces in memory encoding and recall.

## A Plausible Model for the Encoding of Temporal/Spatial Information in Memory Trace Cell

Synchronized oscillations, such as, theta oscillation, gamma bursts or sharp wave ripples (SWR) could coordinate spike times, regulating information transmission and/or carrying information in the temporal domain (Mainen and Sejnowski, 1995; Cardin et al., 2009; Sohal et al., 2009; Shirvalkar et al., 2010; Jadhav et al., 2012; Valero et al., 2015). How could the specific memory trace neurons trigger a temporal pattern of neural activities? Previously, Jadi and Sejnowski (2014) have proposed a simplified model, showing that the frequency and power of the synchronized oscillations, and population spike times, could be modulated by differential synaptic inputs to an Excitation–Inhibition (E–I) balanced circuit. This model consists interconnections between a pair of inhibitory and excitatory neurons and synaptic inputs to them. It is a simplified model but with reasonable biological correlates, as the inhibitory neurons and excitatory neurons are highly wired and likely form reciprocal projections in neocortex (Fino and Yuste, 2011; Gentet et al., 2012; Pala and Petersen, 2015). Such a simple model is intriguing, as it conveys the spatial information into temporal information, where the ratio of synaptic input to two types of neurons (spatially encoded) determines the frequency and power of the network oscillation (temporally encoded). Thereby, while the engram network has been modulated by the history of activities to encode the information, a sustained neural activity from the activated engram cell would go through such a spatially organized network and will trigger the expression of a temporally organized oscillatory activity and deliver it to their targets.

**FIGURE 2:**
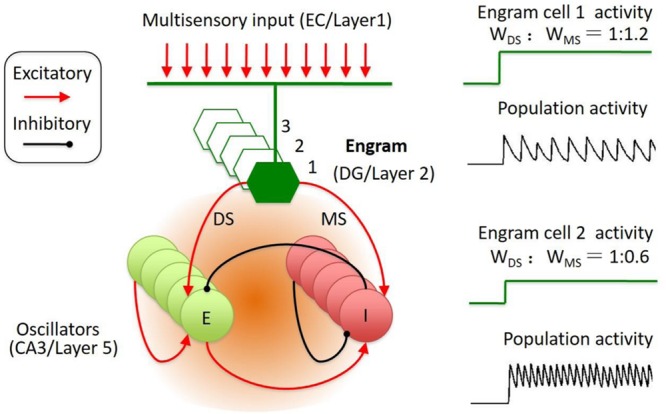
**Engram cell circuit for the representation of spatial–temporal coded information.** The engram cell circuit was inspired by Jadi’s model. The model network architecture featured both excitatory (E) and inhibitory (I) neurons, with recurrent connections between and within E and I populations. In addition, the memory engram cell population sent inputs on both E and I with specified synaptic weights, including di-synaptic and mono-synaptic connection to the inhibitory neuron. The engrams in DG receive input from EC. The memory traces in cortical layer 2 might receive input from layer 1. The reactivation of engram cell is sparse and could trigger the population oscillation at different frequency and amplitude.

Intrigued by this model, we propose that the activities in sparse memory trace neurons are able to initiate a temporally organized oscillatory activity in the downstream network. In this model, a specific memory trace cell is connected to both the excitatory neuron and the inhibitory neuron in a reciprocally connected neural network. Thus, the activity of the trace neurons transmit to inhibitory neuron through the mono- and di-synaptic connections (**Figure 2**). As the synaptic strength ratio of the mono-and-di-synaptic connections to inhibitory neurons determines the property of the oscillatory activity (Whittington et al., 1995; Ermentrout and Kopell, 1998; Brunel and Wang, 2003; Jadi and Sejnowski, 2014), the activation of the trace cell triggers the expression of a specific oscillatory activity. In this case, activation of memory trace cell is able to engage any selective oscillation pattern to present the temporally encoded information in the downstream targets.

This simplified model could be an abstract from two brain regions. In the neocortex, layer 2 neurons were identified as memory traces. In hippocampus, DG neurons were identified as memory trace cells (Tonegawa et al., 2015a). The downstream targets of the memory trace neurons are the oscillators (E–I balanced circuit), such as the CA3 neurons in hippocampus and the layer 5 neurons in neocortex (**Figure 2**). The memory trace cells were sparsely activated in both hippocampus and neocortex (**Figure 1**). The sparse activities of trace cells might set a high threshold to distinct different sensory inputs, thereby, reducing the noises of sensation. Thus, the sparsity of the activities in memory trace neurons ensures the precision of representation for the temporally coded information in their downstream targets.

## Selective Consolidation of Oscillatory Activity into the Specific Memory Trace Cells

The identified memory traces located in specific areas in the brain. Cortical memory traces were identified in superficial layer 2 (Cowansage et al., 2014; Xie et al., 2014), which is very close to layer 1 and receive major inter-cortical connections from multiple cortical areas. The layer 2 neurons receive sensory inputs from layer 4 and top-down or cross-model inputs from layer 1 (Petersen and Crochet, 2013). Thereby, the activation of memory engram cells is modulated by population activity of local layer 4 neurons with a unique temporal–spatial organization. Such network might implicate the transformation of temporally organized activities into the specific activities of spatially distinguished memory trace neurons.

**FIGURE 3:**
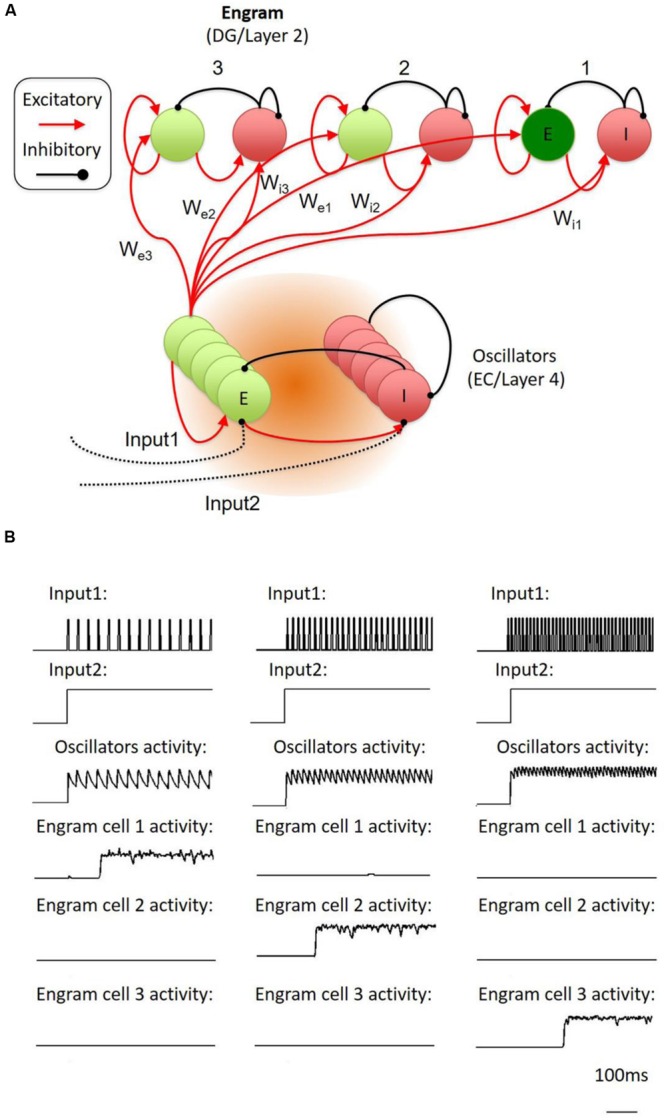
**Proposed temporal-to-spatial transformation in the engram cell circuit. (A)** The whole system was inspired by Jadi’s model. The model contains an oscillator part and three memory engram parts. Each of them was made up of interconnected inhibitory and excitatory neurons. Input1 was an oscillatory stimulation to oscillator’s inhibitory neuron through di-synaptic pathway. Input2 was a constant simulation to oscillator’s inhibitory neuron through mono-synaptic pathway. When the strength of the peak of Input1 was lifted to higher enough, it generated a synchronized oscillation in the oscillator part. The weights of the connection from oscillators’ excitatory neurons to different part of engram neurons (*W*_e1_, *W*_e2_, *W*_e3_) and different part of inhibitory neurons (*W*_i1_, *W*_i2_, *W*_i3_) were different from each other. **(B)** The oscillator was stimulated with different frequency oscillatory input (input1). If the oscillations exactly suit the weights from the oscillator to the part contained the specific engram neuron population and inhibitory neurons, the specific engram neuron population will fire in a very short delay (about 200 ms), Here, we showed that engram cell population 1, 2, and 3 followed low, medium, and high oscillation, respectively.

In fact, the conversion of temporally organized oscillatory activities into the activation of a specific neuron population is a reversed version of the memory trace-to-oscillator network. By engineering the inhibitory-excitatory circuit, the network is able to establish the specific conversion of activity from temporal domain into spatial domain. To test the possibility of such conversion, we proposed a model of cortical network, which is made up of stochastic spiking neurons as oscillator and memory engram cell populations (**Figure 3A**). According to the simulation on this model, oscillatory activities at different frequencies will trigger the activation of a specific engram cell population, which is connected to the oscillator with specified weights (**Figure 3B**; see Supplementary Material for details). Thus, the inhibitory-excitatory circuit might underlie the conversion of neural activities between the temporal domain and the spatial domain, allowing the memory trace cell circuit to conduct encoding and recall of the temporal–spatial information.

## Conclusion

Memory engram cells are specific neuron populations, which are activated by a particular event or a context. Reactivation of those cells is closely related to the memory recall. Artificial reactivating of memory engram cells triggered selective memory-related behavior consequences, while selective lesion of memory traces rendered the memory recall. Such facts demonstrate the critical role of the memory traces as the hub component of memory circuit in the central nervous system.

By now, most of the studies are still focusing on the spatial domain of the memory circuit, leaving an unknown question for the encoding and recall of temporally organized activities. Although still lack of biological evidences, here, we proposed a network model trying to interpret the dynami cs of the memory circuit. While the memory engram cells are located in physically wired circuit, dynamic activities with distinct temporal properties are the results of the activities of specific memory engram cells. The model for the conversion of neural activities between the temporal and the spatial domains implicates that it is possible for the memory engram cells and their neural networks to encode spatially and temporally organized information in form of synaptic changes of the inhibitory–excitatory circuit. Furthermore, the activation of memory engram cells might engage the modified circuit to excite temporally and spatially organized activities, representing the recall of the memory. Although this model is simple and attractive, other possibilities might also remain. Nonetheless, we provided a plausible solution here to argue that sparse engram cells are capable to induce representation of complex information, containing both temporal and spatial domains. Such sparse responses of memory storage provide a new avenue into the circuit mechanism of memory encoding and recall.

## Author Contributions

J-SG, JJ, and HX wrote the article and generated the figures. K-YL proposed and simulated the model.

## Conflict of Interest Statement

The authors declare that the research was conducted in the absence of any commercial or financial relationships that could be construed as a potential conflict of interest.
