# Supplementary material for: How Does the Sparse Memory “Engram” Neurons Encode the Memory of a Spatial–Temporal Event?
Source: Front Neural Circuits. 2016 Aug 23;10:61. doi: 10.3389/fncir.2016.00061 (PMC4993949; doi:10.3389/fncir.2016.00061)
Supplement: Supplementary file 1 [file Data_Sheet_1.DOCX]

**Appendix**

The model shown in Figure 3 was built up of stochastic spiking neuron models. The single neuron model is two-state Markov processes. We choose 1 ms as the time step in our simulation (see Jadi’s model for details). There are two parts in the model: the major oscillator and three small engram oscillators. Each of the oscillator is similar to the Jadi's model. There are N_E_ = 800 excitatory and N_I_= 200 inhibitory neurons in the major oscillator and N_E_ = 80 excitatory and N_I_= 20 inhibitory neurons in each of engram oscillator. Each engram oscillator was connected to the major oscillator with given connections of W_e_ and W_i._. The averaged activities in the excitatory neurons of one engram oscillator were calculated upon the input of different oscillatory activities from the input 1. Selective responses were observed between engram oscillators.

Oscillators are all-to-all neuron networks. The weights within the oscillator are given by follows:

E-to-E:$w_{EE}=\frac{W_{EE}}{N_{E}}$

I-to-E:$w_{EI}=\frac{W_{EI}}{N_{I}}$

E-to-I:$w_{IE}=\frac{W_{IE}}{N_{E}}$

I-to-I:$w_{II}=\frac{W_{II}}{N_{I}}$

We took W_EE_ = 16, W_EI_ = 26, W_IE_ = 20, and W_II_ = 1, similar to the model of previously described one. Input2 was set as 16. Input1 was set as

$$Input1\left( t \right)=\left\{ \begin{aligned} 30,for t\equiv0,1,2(mod T) \\ 0,for the rest t \end{aligned} \right.$$

T is the numerical period of Input1. The network was tested under three frequencies. T=33ms, f=1000/33=33.33Hz; T=18ms, f= 1000/18=55.56Hz; T=12ms, f=1000/12=83.33Hz. Inhibitory neurons in the engram oscillator received constant inhibitory input as 499.5/173.5. The weights between excitatory neuron and engram oscillator were as follows:

W_e1_ (t)=512/600, W_i1_ (t)=512/632;

W_e2_ (t)=512/645, W_i2_ (t)=512/665;

W_e3_ (t)=512/680, W_i3_ (t)=512/720.
